# Highly-Efficient and Visible Light Photocatalytical Degradation of Organic Pollutants Using TiO_2_-Loaded on Low-Cost Biomass Husk

**DOI:** 10.3390/ma15238671

**Published:** 2022-12-05

**Authors:** Yuan Li, Xirong Lin, Zhanpeng Li, Jinyun Liu

**Affiliations:** 1 Sichuan Vocational and Technical College, Suining 629000, China; 2 National Key Laboratory of Science and Technology on Micro/Nano Fabrication, Department of Micro/Nano-Electronics, Shanghai Jiao Tong University, Shanghai 200240, China; 3 Nanjing Noland Environmental Engineering Technology Co., Ltd., Nanjing 211215, China; 4 Key Laboratory of Functional Molecular Solids, Ministry of Education, College of Chemistry and Materials Science, Anhui Normal University, Wuhu 241002, China; 5 Anhui Key Laboratory of Molecule-Based Materials, College of Chemistry and Materials Science, Anhui Normal University, Wuhu 241002, China

**Keywords:** TiO_2_, rice husk, photodegradation, methyl orange, ecological restoration

## Abstract

A composite composing of TiO_2_ nanoparticles load on biomass rice husk (RH) is developed by directly growing TiO_2_ nanoparticles on RH. The in-situ growth of the nanocrystals on RH is achieved by a low-cost and one-step homogeneous precipitation. Rapid hydrolysis proceeds at 90 °C by using ammonium fluotitanate and urea to facilitate the selective growth of TiO_2_. The method provides an easy access to the TiO_2_-RH composite with a strong interaction between TiO_2_ nanoparticles and the underlying RH. The structure and composition of TiO_2_-RH are characterized by using X-ray diffraction, X-ray photoelectron spectroscopy, Fourier-transform infrared spectroscopy, and UV-vis absorption spectroscopy. TiO_2_ nanoparticles-RH exhibits a good photocatalytic degradation of methyl orange. The results show that 92% of methyl orange (20 mg L^−1^) can be degraded within three hours in visible light. The catalytic activity of the composite is not reduced after 6 cycles, and it still reaches 81% after 6 cycles. The enhanced performance is ascribed to the suitable particle size the good dispersibility. It is expected that the high photocatalytical performance and the cost-effective composite presented here will inspire the development of other high-performance photocatalysts.

## 1. Introduction

Photodegradation of organic pollutants has received many interests during the past decades [1]. Titanium dioxide is distinguished and widely studied as a semiconductor photocatalysis [2,3]. It has been considered a promising catalyst because of its non-toxicity, cheapness, stability and high reactivity [4], and it has been used for removing environmental pollutants in air or water [5,6]. In recent years, organic/inorganic composites are used as catalysts, sensors, etc [7,8,9]. Some reports display that the TiO_2_-based composites show improved catalytic activities [10,11,12,13].

Methyl orange (MO) is a common acid-anionic mono-azo dye which contains an azo group (-N=N-). MO is used widely in printing and dyeing industries. The release of MO is harmful to the environment. As a result, the removal of MO in the industrial waste water has been of the most important tasks. As a represented azo dye, MO has been employed as a simulated target for photocatalytical measurements [14].

Biomass waste is a kind of cheap and easily available natural organic resource. Rice husk (RH) is composed of cellulose (34–46%), pentose (21–22%), lignin (9–20%), and protein (2–3%), which is an abundant agro-industrial residue [15,16]. However, there is still a considerable amount that cannot be used rationally, causing serious environmental problems. RH with tubular structure has a large specific surface area, rich resources, ideal chemical stability and robust strength. These characteristics enable RH to be used to remove heavy metal ions in aqueous solutions [17,18]. There is a layer of SiO_2_ on the outer surface of rice husk as a protective film for natural RH [19]. By using the alkali treatment, SiO_2_ can be removed, so that some functional groups could be exposed on the surface of RH. In addition, the ester bond between cellulose and lignin can be opened, and part of the cellulose and lignin can be dissolved to increase the loading of TiO_2_, and thus, enhancing the catalytic activity of TiO_2_-RH composite.

Despite a good photocatalytic activity of granular TiO_2_ nanoparticles, their recycling and reuse limit their application in water treatment, leading to considerable loss [20,21,22]. Due to relatively mild catalytic conditions, moderate catalytic activity, environmental friendliness, and relatively low biological toxicity, TiO_2_ photocatalyst has become an ideal choice to remove organic pollutants in air and water [23,24]. Although TiO_2_ nanoparticles have high scientific research value, the size of their particles limits their practical application in water treatment. Considering this, it is relatively difficult to recover and reuse them, and the synthesis cost is high. Moreover, the complicated environment in water is easy to interfere with the surface properties of TiO_2_, which limits their applications in water treatment [25].

In order to enhance the reuse capability of TiO_2_, RH is used as a substrate to load TiO_2_. The RH loaded with TiO_2_ nanoparticles can contact well with pollutants in water and achieve a good photocatalytic activity of the TiO_2_-RH composite. Here, TiO_2_-RH composite is prepared through a co-precipitation approach, which is used to decompose MO in visible light. The developed preparation method and the prepared photocatalyst would possess the following features: (i) The preparation method is quite simple. A low-temperature one-step approach for preparing anatase TiO_2_ reduces the preparation cost, which is significant for potential applications. (ii) TiO_2_ photocatalyst has many applications in the treatment of exhaust gases; however, it is restricted due to the potential biotoxicity when the particle size is non-ideal. In our study, the particle size is able to be controlled easily at around 100 to 300 nm which has a low biotoxicity, and it is beneficial for real use. (iii) The TiO_2_-RH developed here displays a high photocatalytical performance due to the suitable particle size the good dispersibility. On the basis of an optimized amount of RH and and the cycling experiments on the TiO_2_-RH photocatalyst, it maintains a high catalytic performance after six rounds of repeated degradation, indicating a potential commercializing value. Considering some previous reports [26], the TiO_2_-RH composite presented here is able to degrade 92% of methyl orange (20 mg L^−1^) within three hours in visible light, which is competitive compared to some other photocatalysts.

## 2. Experimental

### 2.1. Preparation of Composite

RH was purchased from Nanjing. China. (NH_4_)_2_TiF_6_ (analytical grade) was obtained from Aladdin industrial Co., Ltd. (HongKong, China). NaOH, CO(NH_2_)_2_ and MO (analytical grade) were obtained from Tianjing Kemiou Chemical Reagent Co., Ltd. After being broken and sieved, the RH was added in to a 0.5 mol L^−1^ of NaOH solution. The solution was kept at 120 °C for 8 h. Then, RH was washed to neutral, then dried at 80 °C. A series of amounts of RH were added to the (NH_4_)_2_TiF_6_ and CO(NH_2_)_2_ solutions with stirring. The mixture was heated to 90 °C under stirring for 60 min. The final product was collected by centrifuge, washed with deionized water, and then dried at 80 °C. Samples with different contents of RH (0.5, 1.0, 1.5, 2.0, and 2.5 g) were prepared to investigate the effect of RH on the structure and photocatalytical performance. For example, TiO_2_-2RH represents 2 g of RH was added to load with TiO_2_ nanoparticles.

### 2.2. Characterization

The prepared TiO_2_-RH was characterized by a Tongda TD-3500 X-ray powder diffractometer (XRD, Tonda S&T Co., Ltd., Dandong, China), field emission scanning electron microscopy (FE-SEM, FEI company, USA), Hitachi S4800 scanning-electron microscope (Hitachi Company, Japan), Thermo Scientific Nicolet Fourier-transform infrared spectra (FTIR, Thermo Scientific Company, USA), and Kratos XSAM X-ray photoelectron spectra (XPS, Kratos Company, Manchester, England). In addition, UV-Vis spectra were obtained using a TU-1901 spectrophotometer (Dingnuo Co., Ltd., Shanghai, China).

### 2.3. Photocatalytic Evaluation

To investigate the photocatalytic activity of the TiO_2_-RH, MO was used in a circulating water-cooling beaker with magnetic stirring. Photocatalysts (100 mg) were added in 100 mL of MO solution (20 mg L^−1^) and stirred for 20 min before irradiation. Samples were taken at 30 min intervals during degradation. A 350 W high pressure xenon lamp, which emits a similar spectrum as sun, was used as the visible light source, which was positioned 20 cm away from circulating water-cooling beaker. The concentration of MO was measured at 464 nm using spectrophotometer.

## 3. Results and Discussion

**Figure 1:**
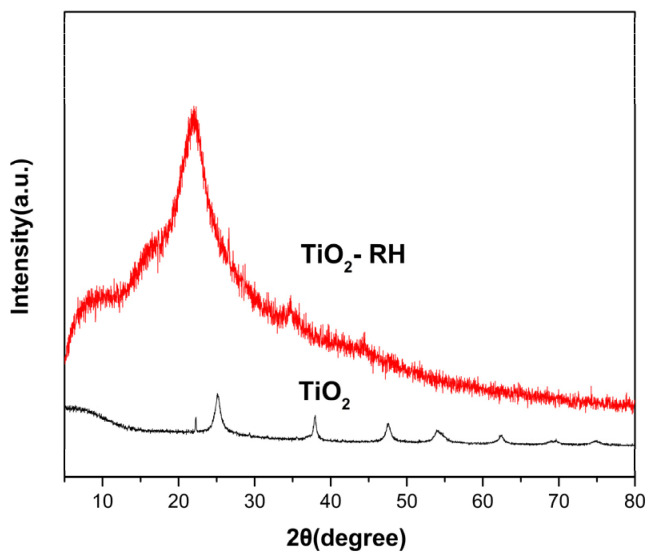
XRD patterns of TiO_2_ and TiO_2_-RH.

The XRD patterns of the pure TiO_2_ and TiO_2_-2RH are presented in Figure 1. According to the diffraction peaks of typical anatase TiO_2_ (JCPDS card No. 01-071-1166), the TiO_2_ without RH shows an anatase phase of titanium dioxide. The diffraction peak of TiO_2_-2RH at 22.5° in the sample is caused by cellulose in the synthesized material [11,12,13]. The characteristic peak of TiO_2_ is not observed in the sample of TiO_2_-2RH, probably because the characteristic peak of TiO_2_ is covered by the signals of RH [27].

**Figure 2:**
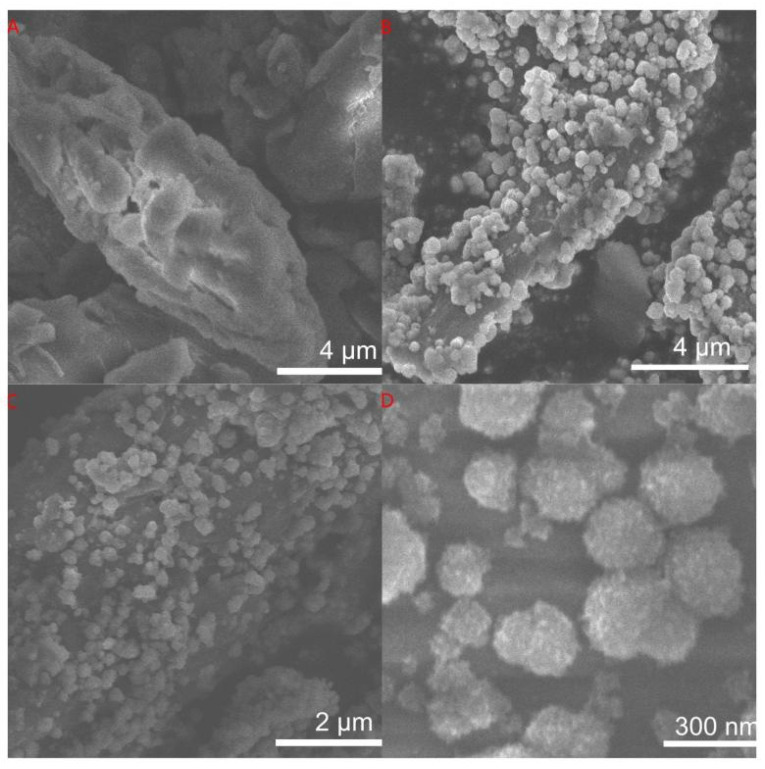
SEM images of the (**A**) RH and (**B**–**D**) TiO_2_-2RH.

Figure 2 displays the SEM photographs of RH and TiO_2_-RH composite. The TiO_2_-RH shows highly dispersed submicron-scale anatase TiO_2_ spheres with a layered structure. Each TiO_2_ microsphere has a hierarchical structure, indicating that the microsphere contains several TiO_2_ nanoparticles. The special structure of TiO_2_ microspheres indicates that it is formed by the aggregation of TiO_2_ nanoparticles on RH. SEM images show that dense TiO_2_ nanocrystals are assembled on the RH surface. TiO_2_ nanoparticles remain on the surface of the RH even under prolonged sonication, indicating a robust structure. The prepared TiO_2_-RH composite is submicron, and the catalyst can be separated from the degraded object solution by simple filtration after the photocatalytic reactions. In Figure 2D, the SEM images shows that the particle size is between 100–300 nm, which is well controllable by changing the reaction time [23,24].

**Figure 3:**
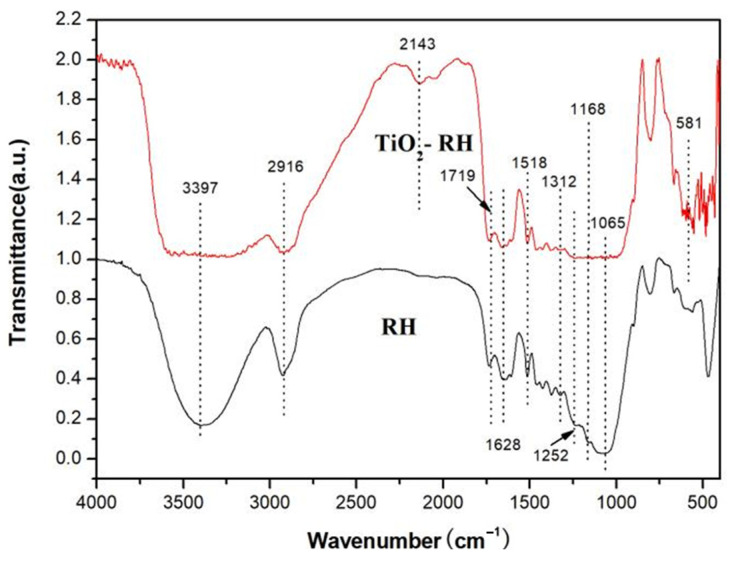
FTIR spectra of RH and TiO_2_-RH.

In Figure 3, FTIR spectra of RH and TiO_2_-RH composite are presented. Vibration absorption band of TiO_2_-RH composite between 1400 and 450 cm^−1^ belongs to characteristic mode of TiO_2_. The bands at 610 and 1390 cm^−1^ are ascribed to Ti−O and Ti−O−Ti stretching, respectively [11,12,13]. Strong −OH stretching (3300–3500 cm^−1^) in TiO_2_ and TiO_2_-RH composite may be due to the adsorbed water [12]. FT-IR spectrum of RH presents a vibrational band at 2916 cm^−1^, which is assigned to C−H stretching; while the band at 1628 cm^−1^ is indexed to C=C stretching. The band between 1387–1016 cm^−1^ may be caused by C−O stretching and C=C bending, and such vibrational peaks mostly belong to cellulose, lignin, and biopolymers [11].

**Figure 4:**
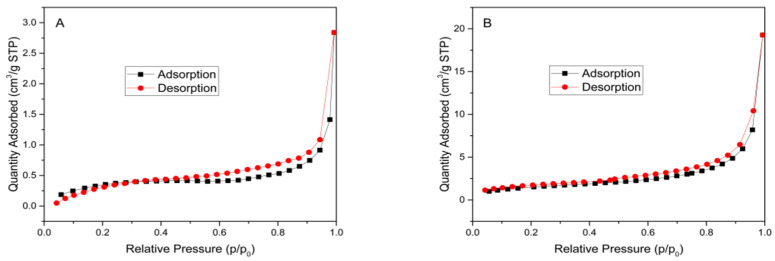
Adsorption-desorption profiles of (**A**) RH and (**B**) TiO_2_-RH.

In Figure 4, both the specific surface area and pore volume of the synthesized TiO_2_-RH composite increase, while the average pore-size decreases. In other words, the improvement of the catalytic performance of the composite is also related to the increase in the specific surface area, as shown in Table 1.

**Figure 5:**
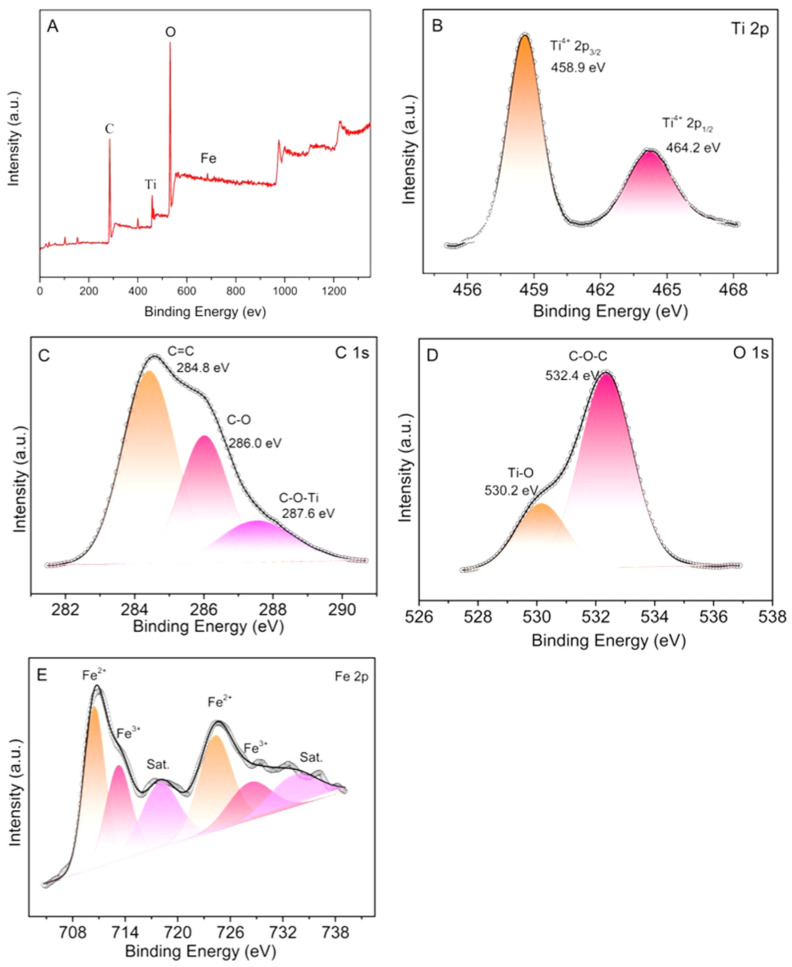
XPS spectra of the TiO_2_-RH composite: (**A**) Survey spectrum, (**B**) Ti 2p, (**C**) C 1s, (**D**) O 1s, and (**E**) Fe 2p.

In addition, we conducted XPS measurements on the TiO_2_-RH composite. In Figure 5, it can be seen that there are mainly titanium, carbon, oxygen, iron, and other elements in the composite. In the Ti 2p spectrum, the peaks at 458.9 and 464.2 eV correspond to Ti 2p3/2 and Ti 2p1/2, respectively, which further verifies the existence of Ti^4+^ oxidation state in TiO_2_-RH. According to Figure 5C, C-O-Ti exists in TiO_2_-RH composite, which proves that TiO_2_ crystal and RH are combined in the form of chemical bond. The two peaks of the O 1s at 530.2 and 532.4 eV correspond to lattice oxygen and C−O in RH. Figure 5E shows the presence of an oxide of Fe, which may be introduced by the shredder during rice-husk shredding [27,28].

**Figure 6:**
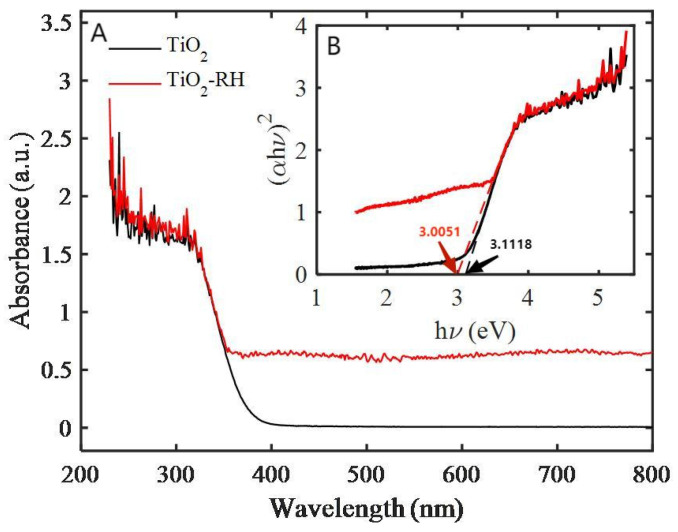
(**A**) UV–vis absorption spectra, (**B**) Tauc’s profile for the band-gap determination.

Figure 6A shows the UV-vis absorption spectrum of TiO_2_-RH composite. As expected, the characteristic peak of TiO_2_ nanocrystals is around 400 nm, and there is almost no absorption of light higher than 400 nm. However, it is interesting to note that the TiO_2_-RH composite exhibits an unusual UV-visible absorption when the TiO_2_ is loaded on RH. The UV-vis absorption peak of the TiO_2_-RH composite has a redshift relative to the TiO_2_ nanoparticles. Moreover, the amount of RH also affects the optical properties of TiO_2_-RH. Wavelength of light absorbed by the composite almost covers the entire UV-visible region. This phenomenon is consistent with the optical properties of carbon-doped anatase TiO_2_. TiO_2_-RH composite can absorb more light than single TiO_2_ nanoparticles, indicating that the composite would be able to improve the absorption of visible light, which may also change the process of generating electron-hole pairs in visible light [13]. On the basis of these mechanism, this unique structure of TiO_2_-RH composite exhibits an improved photocatalytic activity in visible light. Figure 6B shows that the band-gap energy of TiO_2_-RH composite is lower than that of pure TiO_2_, which may be related to the combination of TiO_2_ and rice husk in the composite system [25,28].

**Figure 7:**
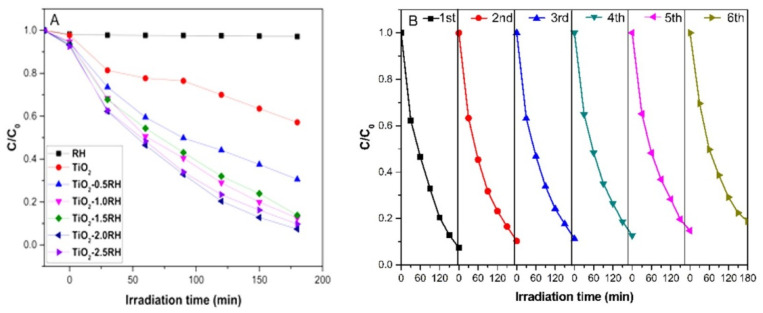
(**A**) Photodegradation kinetics of TiO_2_-RH towards 20 mg L^−1^ MO solution in visible light. (**B**) Cycling photodegradation of MO by TiO_2_-2RH under visible light.

The degradation of MO by TiO_2_ and TiO_2_-RH composites under visible light was investigated, as displayed in Figure 7A. TiO_2_ without RH exhibits a weak photocatalytic activity and degradation rate of MO after 3 h is about 43%. Pure RH displays no capability of photodegradation. In contrast, for the TiO_2_-RH samples with RH = 0.5, 1.0, 1.5, 2.0, and 2.5 g, the degradation rates are 69.4%, 87.4%, 86.2, 92.6%, and 90.3%, respectively. With a small amount of RH, the low catalytic activity is ascribed to the accumulation of TiO_2_ nanoparticles on the surface of RH, which are not well dispersed. Depending on the increase of RH content, the catalytic activity is improved. However, if the amount of RH increases further, the decrease in photocatalytic activity may be caused by the decrease in TiO_2_ content in the composite. The catalytic performance of TiO_2_-RH composite is improved compared with TiO_2_ under visible light; this may be because TiO_2_-RH composite has a certain light absorption in visible light region, while TiO_2_ has very less absorption in visible light. As can be seen from the XPS spectrum, the existence of C-O-Ti bond in TiO_2_-RH composite indicates that TiO_2_ and RH are combined by chemical bond, rather than van der Waals force, which is more conducive to the separation and transmission of electrons. With the increase of RH amount, the catalytic performance is enhanced, possibly because TiO_2_ is more dispersed on the RH surface rather than agglomeration. When the amount of RH reaches 2 g, the catalytic performance is the highest. If the amount increases continuously, the catalytic performance decreases, possibly because the amount of active TiO_2_ catalyst is reduced. TiO_2_ crystals synthesized by co-precipitate method are spherical in shape, and the binding with RH is in the form of C-O-Ti. Therefore, the key factor affecting the catalytic performance would be the effective dispersion of TiO_2_ on the surface of RH. In the prepared TiO_2_-RH, it exhibits a reduced agglomeration, which enables a good catalytic performance compared to the pure TiO_2_ nanoparticles [29].

Figure 7B shows TiO_2_-2RH composite cyclically degrading MO (20 mg L^−1^) in visible light for 180 min per cycle. The catalytic activity of the composite is not significantly reduced after 6 cycles, and it still reaches 81% after 6 cycles. The decrease in the activity of the photocatalyst may be due to the partial shedding of the loaded TiO_2_ nanoparticles.

## 4. Conclusions

In summary, a composite based on RH loading with dense TiO_2_ nanoparticles is developed by a co-precipitation method. The developed preparation method is quite simple. Low-temperature one-step approach for preparing anatase TiO_2_ reduces the preparation cost, which is significant for potential applications. TiO_2_ photocatalyst has many applications in the treatment of exhaust gases; however, it is restricted due to the potential biotoxicity when the particle-size is non-ideal. In our study, the particle-size is able to be controlled easily at around 100 to 300 nm which has been considered has a low biotoxicity for use. Moreover, the TiO_2_-RH developed here displays a high photocatalytical performance due to the suitable particle-size the good dispersibility. The composite shows a good potential in the photocatalytic degradation of MO in visible light. The results show that 92% of MO can be degraded within three hours in visible light. A good cycling photodegradation performance for six rounds is also achieved. It is expected that some other highly-efficient TiO_2_ composite photocatalysts can also be obtained from agricultural biowastes, such as straw, wheat bran, corn cobs, and sawdust. This work converts agricultural biomass waste into a high-performance photocatalyst, and exhibits a promising application for in wastewater treatment. In addition, it is considered that the degradation efficiency of TiO_2_ under visible light would be further improved by doping metal ions and introducing single-atom catalyst, which would be promising research directions.

## Figures and Tables

**Table 1:** Surface parameters of RH and TiO_2_-RH.

Sample	Specific Surface Area (m^2^ g^−1^)	Pore Volume (cm^3^ g^−1^)	Average Pore Diameter (nm)
RH	1.0542	0.004392	11.2855
TiO_2_-RH	4.9642	0.029811	6.5425

## Data Availability

Not applicable.
